# Ассоциация витилиго с эндокринными аутоиммунными заболеваниями: обзор литературы

**DOI:** 10.14341/probl13684

**Published:** 2026-09-08

**Authors:** М. Р. Рагимов, А. А. Юзбекова, А. В. Молочков, И. В. Мисникова

**Affiliations:** Московский областной научно-исследовательский клинический институт им. М.Ф. Владимирского Россия; Moscow Regional Research and Clinical Institute Russian Federation; Центральная государственная медицинская академия Россия; Central State Medical Academy Russian Federation; Московский областной научно-исследовательский клинический институт им. М.Ф. Владимирского; Федеральное государственное автономное образовательное учреждение высшего образования Первый Московский государственный медицинский университет им. И.М. Сеченова Минздрава РФ Россия; Moscow Regional Research and Clinical Institute; Federal State Autonomous Educational Institution of Higher Education I.M. Sechenov First Moscow State Medical University of the Ministry of Health of the Russian Federation Russian Federation

**Keywords:** витилиго, аутоиммунный сахарный диабет, эндокринные заболевания, меланоциты, vitiligo, autoimmune diabetes mellitus, endocrine diseases, melanocytes

## Abstract

Витилиго — дерматологическое заболевание, проявляющееся очаговым исчезновением пигмента меланина на кожных покровах и слизистых оболочках человека. Несмотря на разнообразие предложенных гипотез развития данной патологии, центральная роль принадлежит именно аутоиммунному механизму поражения меланоцитов.

Анализ результатов многочисленных международных исследований подтверждает выраженную тенденцию к увеличению распространенности эндокринных аутоиммунных заболеваний (АИЗ) среди больных витилиго по сравнению с общей популяцией. К таким заболеваниям относятся, прежде всего, аутоиммунный тиреоидит (АИТ), болезнь Грейвса, сахарный диабет 1 типа (СД1) и первичная надпочечниковая недостаточность (ПНН).

Ассоциация многих из этих АИЗ, включая витилиго, с определенными гаплотипами HLA коррелирует с тем, что они входят в состав нескольких типов аутоиммунного полигландулярного синдрома (АПС). Отдельные исследования подтверждают высокую частоту сочетания витилиго с АПС 2, 3 и 4 типов.

Настоящий обзор направлен на систематизацию имеющейся научной литературы относительно частоты встречаемости и особенностей ассоциированных аутоиммунных эндокринных нарушений у лиц, страдающих витилиго.

## Abstract (en)

Vitiligo is a dermatological disease characterized by focal disappearance of the melanin pigment on the skin and mucous membranes of a person. Despite the variety of proposed hypotheses for the development of this pathology, the central role belongs to the autoimmune mechanism of melanocyte damage.

Analysis of the results of numerous international studies confirms a pronounced tendency towards an increase in the prevalence of endocrine autoimmune diseases among patients with vitiligo compared to the general population. Such diseases include, first of all, autoimmune thyroiditis, Graves' disease, type I diabetes mellitus and autoimmune adrenal insufficiency. Some studies confirm a high frequency of vitiligo combination with multiendocrine autoimmune syndromes.

This review is aimed at systematizing the available scientific literature on the frequency of occurrence and characteristics of associated autoimmune endocrine disorders in individuals suffering from vitiligo.

## ВВЕДЕНИЕ

Витилиго — это хроническое аутоиммунное заболевание, характеризующееся потерей пигментных меланоцитов в эпидермисе [1]. Заболевание встречается примерно у 0,5–2% взрослых и детей во всем мире и проявляется в виде амеланотических, молочно-белых, четко очерченных пятен или очагов, окруженных нормальной кожей [2][3]. Депигментация, характерная для витилиго, вызвана прогрессирующим разрушением меланоцитов [4]. Одним из ключевых вопросов, привлекающих особое внимание, является ассоциация витилиго с аутоиммунными заболеваниями эндокринной системы, частота которых может достигать 20–23% [5]. Данные последних десятилетий показывают, что пациенты с витилиго подвержены повышенному риску развития таких серьезных состояний, как заболевания щитовидной железы (в том числе аутоиммунный тиреоидит и болезнь Грейвса), сахарный диабет (СД) и аутоиммунная надпочечниковая недостаточность [6].

Актуальность исследования определяется растущим числом случаев связи витилиго с аутоиммунными заболеваниями эндокринной системы, что придает особую остроту проблеме диагностики и ведения пациентов с данной патологией. Витилиго само по себе является серьезным эстетическим дефектом, приводящим к снижению качества жизни больного [7][8], а ассоциация с серьезными эндокринными заболеваниями создает дополнительные сложности для практикующих врачей разных специальностей.

Принимая во внимание отсутствие четкого понимания молекулярно-биохимических механизмов, лежащих в основе совместного развития витилиго и аутоиммунных эндокринных заболеваний, становится очевидна необходимость глубокого анализа существующей литературы. Понимание природы указанной ассоциации крайне важно для разработки рациональных подходов к диагностике, профилактике и лечению, позволяющих повысить уровень медико-социальной адаптации пациентов.

Данная обзорная статья призвана привлечь внимание широкой медицинской общественности к актуальным вопросам современной науки и практики, стимулируя дальнейшие исследования и внедрение новых стандартов диагностики и лечения.

В ходе анализа были использованы публикации из специализированных научных баз, включая PubMed/MEDLINE, CochraneLibrary, Embase, IndexMedicus и российские профильные журналы по соответствующей тематике, а также актуальные Клинические рекомендации Министерства здравоохранения Российской Федерации по диагностике и лечению витилиго.

## АУТОИММУННЫЙ ПОЛИГЛАНДУЛЯРНЫЙ СИНДРОМ

Витилиго входит в аутоиммунный полигландулярный синдром 2 типа (АПС-2), 3 типа (АПС-3) и 4 типа (АПС-4), которые включают в себя множество иных эндокринных и неэндокринных заболеваний [9][10].

Современное понимание АПС, в частности АПС-2, подтверждает, что витилиго является одним из характерных и часто встречающихся ассоциированных проявлений наряду с гнездной алопецией, целиакией и пернициозной анемией [11][12]. Более того, общность аутоиммунных заболеваний, входящих в комплекс АПС, отражается и в связи с одними гаплотипами группы антигенов гистосовместимости (HLA). Например, многие заболевания, встречающиеся в рамках АПС-2, ассоциированы с HLA В8, DR3, DR4, DR5. Так, витилиго, СД1, ПНН и АИТ, в свою очередь, ассоциированы с HLA DR4 (DRB1*04) [13].

Витилиго в структуре АПС-2 встречается как одно из частых кожных проявлений, что подтверждается данными исследований: в когорте пациентов с витилиго частота ассоциации с АПС-2 достигает 28% для надпочечниковой недостаточности и 69% для аутоиммунных тиреопатий [14]. Генетическая взаимосвязь между этими заболеваниями обусловлена общими гаплотипами HLA II класса, преимущественно HLA-DR3, DR4 и DR5, которые ассоциированы с потерей иммунологической толерантности к антигенам эндокринных тканей [14]. В частности, при АПС-2 наиболее значимыми являются гаплотипы HLA-B8 и HLA-DR3, выявляемые у 60–70% пациентов [14]. Эти же аллели ответственны за предрасположенность к изолированному витилиго, что объясняет высокую частоту коагрегации заболеваний. Современные исследования подчеркивают, что полигенный риск витилиго, связанный с локусами HLA, совпадает с таковым при других компонентах АПС-2 (например, СД1), формируя общую аутоиммунную «конституцию» [14]. Данные генетические ассоциации согласуются с современной концепцией АПС-2 как полигенного заболевания, при котором комбинация вариантов генов, кодирующих ключевые регуляторные белки иммунной системы (такие как HLA, CTLA-4, PTPN22), приводит к потере иммунологической толерантности и одновременному поражению нескольких органов, включая кожу в виде витилиго [11].

## ЗАБОЛЕВАНИЯ ЩИТОВИДНОЙ ЖЕЛЕЗЫ

Наиболее ярко выраженной и доказанной ассоциацией витилиго являются аутоиммунные заболевания щитовидной железы. Проведенный масштабный метаанализ, охвативший 78 исследований, продемонстрировал следующие закономерности. Пациенты с витилиго имели более высокие шансы развития аутоиммунного тиреоидита (OR=10,39, 95% ДИ=2,43–44,40), гипотиреоза (OR=5,54, 95% ДИ=3,36–9,13), гипертиреоза (OR=4,68, 95% ДИ=1,75–12,50), болезни Грейвса (OR=2,93, 95% ДИ=2,62–3,28), тиреоидита Хашимото (OR=2,12, 95% ДИ=1,92–2,34). Также было обнаружено незначительное, но устойчивое увеличение риска рака щитовидной железы (ОР=1,13, 95% ДИ: 1,02–1,24) [15].

Современные исследования демонстрируют значительную вариабельность распространенности АИЗ ЩЖ среди пациентов с витилиго. Ряд авторов сообщает о высоких показателях коагрегации этих патологий. В российской когорте взрослых пациентов (2022 г.) аутоиммунные тиреопатии (включая тиреоидит Хашимото и болезнь Грейвса) диагностированы у 69% обследованных в эндокринологическом центре, причем у 46% из них выявлены множественные эндокринные аутоиммунные нарушения [14]. Аналогичные данные представлены в систематическом обзоре Abuhalimeh RM (2024 г.), где частота АИЗЩЖ достигала 32,1% при акрофациальной форме витилиго, ассоциированной с антителами к тиреопероксидазе (ТПО) [16][17].

В то же время другие исследования фиксируют более низкую распространенность. Так, в работе Teh et al. (2024 г.) на малайзийской выборке АИЗЩЖ выявлены лишь у 3,2% пациентов [16]. Глобальный метаанализ 2024 г., объединивший 82 230 пациентов, указывает среднюю частоту АИЗЩЖ на уровне 3,5% (диапазон: 3,2–28,7%) [16]. Несмотря на статистические расхождения (от 3,2% до 69%), все исследования подтверждают превышение общепопуляционных показателей.

Витилиго демонстрирует значимую ассоциацию с наличием антител к тиреопероксидазе (АТ-ТПО), являющихся высокоспецифичным серологическим маркером АИТ и предиктором первичного гипотиреоза [6]. Реже, но также выявляется связь с антителами к тиреоглобулину (АТ-ТГ) (дополнительный маркер АИТ) и антителами к рецептору ТТГ (TRAb), характерными для болезни Грейвса [18]. Частота обнаружения АТ-ТПО у пациентов с витилиго достигает 20–40%, что существенно превышает популяционные показатели и подтверждает общие патогенетические механизмы [6].

Cистематический обзор согласно критериям PRISMA [19], выполненный учеными Саудовской Аравии, подтвердил, что нарушение функции щитовидной железы оказывает негативное воздействие на течение витилиго. Например, гипертиреоз способен спровоцировать начало или усиление депигментации, а гипотиреоз замедляет восстановление тканей и снижает эффективность лечебных процедур. В связи с этим, регулярная оценка показателей тиреоидной функции особенно важна для пациентов с витилиго [20]. Эти данные позволяют предположить, что успешное лечение витилиго возможно лишь при условии стабилизации функции щитовидной железы наряду с поддержанием общего здоровья пациента [16].

В контексте патогенеза заслуживают внимания результаты иммуногистохимического анализа, проведенного китайскими исследователями [21]. Ими было установлено, что в образцах ткани щитовидной железы пациентов с аутоиммунным тиреоидитом (АИТ) без сопутствующего витилиго наблюдается экспрессия четырех меланоцитарных антигенов: тирозиназы (TYR), белка 2, ассоциированного с тирозиназой (TRP2), гликопротеина лизосомальной мембраны LAMP1 и CD69. Примечательно, что экспрессия тирозиназы наблюдалась исключительно у пациентов с АИТ и отсутствовала в здоровой ткани щитовидной железы. В то же время антигены TRP2, LAMP1 и CD69 экспрессировались как у больных АИТ без витилиго, так и в нормальной щитовидной железе, однако уровень экспрессии LAMP1 и CD69 был статистически значимо выше в группе пациентов с АИТ (p<0,005). На основе этих данных авторы выдвинули гипотезу о вероятном развитии у пациентов с витилиго иммунных механизмов, способных вызывать вторичное повреждение щитовидной железы с последующим развитием АИТ [6][21].

Согласно крупным когортным исследованиям и метаанализам, распространенность болезни Грейвса среди взрослых пациентов с витилиго варьирует от 0,9% до 17,1% в зависимости от диагностических критериев, географического региона и особенностей когорты. Наибольшие показатели характерны для специализированных клиник Европы, минимальные — для популяционных исследований в Африке и Южной Америке [6][22][24][25]. Крупное исследование Mendelian Randomization (2025 г.) подтвердило двустороннюю причинно-следственную связь между витилиго и БГ (OR=1,14, *p*<0,001). Распространенность БГ у пациентов с витилиго колебалась от 1,8 до 12,6% в зависимости от региона и методов диагностики [22]. Систематический обзор в Dermatology (2024 г.) объединил данные 17 исследований: среди 14 390 взрослых с витилиго частота БГ составила 0,9–15,2%. Самые высокие показатели выявлены в Европе (до 15,2%) и Азии (до 11,7%), самые низкие — в Африке (0,9–3,1%) [23]. В рамках масштабного общенационального исследования, проведенного в Южной Корее, в котором приняли 73 336 человек с диагнозом витилиго и 146 672 добровольцев из контрольной группы, выяснилось, что распространенность болезни Грейвса составляет 0,86% среди больных витилиго и 0,33% в контрольной группе. Ассоциация витилиго и болезни Грейвса чаще всего регистрировалась у молодых людей в возрасте от 20 до 39 лет [24].

Таким образом, учитывая значительный рост заболеваемости и убедительную доказательную базу, рекомендуется проводить ранний скрининг функции щитовидной железы у пациентов с витилиго для предотвращения поздней диагностики и ухудшения состояния в результате развития осложнений.

## САХАРНЫЙ ДИАБЕТ

В последнее время все больше внимания уделяется взаимосвязи между витилиго и СД, в частности потенциальной роли витилиго в развитии инсулинорезистентности и метаболических нарушений.

Эпидемиологические данные, полученные авторами метаанализа литературы, свидетельствуют о высокой вариабельности распространенности СД среди взрослых пациентов с витилиго, которая достигает от 0,1 до 51% в Европе, Америке и Азии, а среди детей эта цифра колеблется от 0,16 до 0,8% [6].

Стоит подчеркнуть результаты важного метаанализа, объединяющего девять крупных исследований с совокупным количеством участников 15 657 человек, страдающих витилиго. Подгрупповой анализ продемонстрировал, что витилиго ассоциировано с повышенным риском развития СД1 (ОШ 2,899, 95% ДИ 1,532–5,482; P=0,001) и сахарным диабетом 2 типа (СД2) (ОШ 2,371, 95% ДИ 1,712–3,283; P<0,001). Установленная корреляция между витилиго и СД1 объясняется общими аутоиммунными механизмами в рамках полигландулярных синдромов [25].

У пациентов с витилиго распространенность СД2 достигает 12% против 6% в контрольных группах, а генетические исследования методом менделевской рандомизации демонстрируют повышение риска витилиго при СД2 (OR=1,13; p=0,043) [26–29]. Согласно исследованию EpiChron Cohort (2025 г.) с участием 218 пациентов с витилиго, у 71,5% выявлена мультиморбидность, включая метаболический синдром (OR=1,78, p<0,014) и СД2. При этом риск СД2 был выше у пациентов с несегментарной формой витилиго и длительностью заболевания >5 лет [30]. Эта ассоциация объясняется общими патогенетическими путями, включая системное воспаление, окислительный стресс и метаболические нарушения.

Ключевую роль играет хроническое системное воспаление, характеризующееся стойким повышением провоспалительных цитокинов (TNF-α, IL-6, IL-1β) и маркеров окислительного стресса, включая малоновый диальдегид и 8-оксо-2›-дезоксигуанозин [31][32]. Данные медиаторы непосредственно влияют на метаболические процессы: TNF-α и IL-6 подавляют сигнальный путь IRS-1/PI3K в гепатоцитах и адипоцитах, нарушая транслокацию GLUT-4 и индуцируя инсулинорезистентность [33]. Параллельно окислительный стресс угнетает функциональную активность ядерного рецептора PPAR-γ — критического регулятора липидного гомеостаза и сенситивности к инсулину. Клинически значимым маркером выступает CXCL10, уровень которого достоверно коррелирует с индексом HOMA-IR (r=0,68; p<0,01), подтверждая взаимосвязь иммунопатологических процессов при витилиго с метаболической дисфункцией [33].

Существенным патогенетическим звеном является дерегуляция меланоцитарного пула в жировой ткани. Меланоциты подкожной и висцеральной жировой клетчатки экспрессируют биологически активные молекулы, включая β-эндорфин (модулятор секреции инсулина) и супероксиддисмутазу (ключевой эндогенный антиоксидант) [33]. При витилиго наблюдается дефицит этих клеток (40–60% снижение, p<0,001), что ведет к накоплению реактивных форм кислорода в адипоцитах, снижению экспрессии глюкозного транспортера GLUT-4 и активации липолиза. Эти изменения инициируют каскад метаболических нарушений: гипергликемию натощак (r=0,72; p=0,003) и компенсаторную гиперинсулинемию (r=0,81; p=0,001) [33].

Митохондриальная дисфункция выступает значимым отягчающим фактором в патогенезе ассоциации витилиго и СД2. У пациентов объективно регистрируется уменьшение числа копий митохондриальной ДНК (на 30%, *p*<0,001) и структурно-функциональные дефекты комплекса I дыхательной цепи, обусловливающие гиперпродукцию супероксида. Данные нарушения инициируют самоподдерживающийся патологический цикл: окислительный стресс индуцирует повреждение митохондриального аппарата, что потенцирует дальнейшее накопление активных форм кислорода. Последующая активация транскрипционного фактора NF-κB стимулирует синтез провоспалительных цитокинов, которые, в свою очередь, угнетают PPAR-γ-опосредованную сигнализацию. Результатом этого каскада является прогрессирование инсулинорезистентности и дислипидемии [31][33]. Дефицит меланоцитов жировой ткани усугубляет указанные процессы посредством снижения β-эндорфин-зависимой стимуляции β-клеток поджелудочной железы и ослабления эндогенной антиоксидантной защиты [33].

Клинически данные механизмы определяют необходимость активного скрининга метаболических нарушений у пациентов с витилиго, включая оценку гликемии натощак, HbA1c и липидного профиля [32][34]. Терапевтические стратегии, направленные на разрыв патогенетических звеньев (ингибиторы JAK-киназ для подавления цитокиновой сети, агонисты PPAR-γ для восстановления инсулиновой чувствительности, антиоксиданты для коррекции оксидативного стресса), демонстрируют потенциал в снижении кардиометаболических рисков в данной популяции [32][33].

## ПЕРВИЧНАЯ НАДПОЧЕЧНИКОВАЯ НЕДОСТАТОЧНОСТЬ

Предполагаемые механизмы, лежащие в основе ассоциации витилиго и надпочечниковой недостаточности, включают общие генетические факторы, аутоантитела, нацеленные как на меланоциты, так и на клетки коры надпочечников, а также дерегуляцию иммунной системы.

Доступные данные о распространенности первичной надпочечниковой недостаточности (ПНН) в европейских странах демонстрируют значительную вариабельность, колеблясь от 93 до 144 случаев на 1 миллион населения, что подчеркивает необходимость дальнейших исследований для точной оценки глобальной распространенности заболевания [35]. Ассоциация витилиго с ПНН. Хотя отдельные исследования указывают на низкую распространенность ПНН при витилиго (0,3%) [36], эти данные статистически ограничены из-за малого числа случаев (n=2). Более репрезентативные работы демонстрируют иные результаты. Так, в российской когорте Петрова В.А. и соавт. (n=61) ПНН выявлена у 4,9% пациентов с несегментарным витилиго, что в 12 раз превышает популяционные показатели (0,0004%) [37]. Аналогично, мультицентровое исследование РНИМУ (2022 г.) зафиксировало ПНН у 28% пациентов в структуре аутоиммунного полигландулярного синдрома 2-го типа (АПС-2) [14]. Важно отметить, что данные других исследователей не выявили существенных различий в распространенности ПНН между больными витилиго и общей популяцией [38], что подчеркивает противоречивость имеющихся данных и необходимость проведения более масштабных исследований для окончательного установления связи между витилиго и надпочечниковой недостаточностью.

## ЗАКЛЮЧЕНИЕ

В свете приведенного обзора актуальной научной литературы мы можем констатировать, что проблема ассоциации витилиго с эндокринными заболеваниями заслуживает отдельного и тщательного рассмотрения. Исследователи последовательно фиксируют наличие значимых статистических зависимостей между витилиго и широким спектром аутоиммунных эндокринных патологий, таких как АИТ, болезнь Грейвса, СД и ПНН.

Современные подходы к изучению патогенеза витилиго постепенно интегрируют различные направления фундаментальной и прикладной медицины. Механизм развития витилиго оказывается связанным как с иммуноопосредованными механизмами повреждения меланоцитов, так и с иными аутоиммунными заболеваниями, включая эндокринные.

Полученные данные ставят перед медицинским сообществом важные практические задачи. Во-первых, необходима реализация последовательных мер по повышению осведомленности врачей всех специальностей о существовании потенциальных ассоциаций витилиго с эндокринными заболеваниями. Во-вторых, врачам следует проявлять повышенное внимание к возможным проявлениям и признакам эндокринных нарушений у пациентов с установленным диагнозом витилиго и проводить соответствующую диагностику в случае наличия подозрительной клинической картины. Такой подход обеспечит своевременное выявление эндокринных заболеваний и позволит начать эффективное лечение, предотвращающее развитие опасных осложнений. Помимо практических выводов, возникает потребность в проведении дальнейших междисциплинарных исследований, направленных на глубокое изучение патогенетических основ взаимосвязи витилиго и эндокринных заболеваний. Определение конкретных биомаркеров, подтверждающих такую связь, станет основой для создания персонализированного подхода к лечению и профилактики данных состояний.

Несмотря на достигнутые успехи в изучении темы, остаются нерешенными вопросы, касающиеся генетики, иммунологии и патофизиологии, необходимые для полного раскрытия сложных взаимоотношений между витилиго и эндокринными заболеваниями. Перспективы будущих исследований включают создание унифицированной базы данных пациентов с витилиго, стандартизацию лабораторных протоколов и международное сотрудничество ученых, работающих в области аутоиммунных заболеваний.

Таким образом, современные данные свидетельствуют о том, что патогенез витилиго включает как аутоиммунные, так и неаутоиммунные механизмы, что объясняет многообразие его проявлений и взаимодействие с различными формами СД.

Современные стандарты терапии витилиго, отраженные в международных руководствах и Клинических рекомендациях Минздрава РФ, претерпели значительные изменения. На смену неспецифическим иммуносупрессантам пришла таргетная терапия. Топические ингибиторы янус-киназ (JAK), в частности руксолитиниб (рукселитиниб), утверждены в качестве терапии первой линии для лечения несегментарного витилиго у взрослых и подростков. В клинической практике также широко применяются и продолжают исследоваться другие JAK-ингибиторы (тофацитиниб, рилтлецитиниб). Классические методы, такие как топические кортикостероиды, ингибиторы кальциневрина и узкополосная средневолновая УФБ-фототерапия (NB-UVB) или эксимерный лазер/лампа, остаются важными компонентами лечения, особенно в комбинации с таргетными препаратами. Подход, основанный на патогенезе, позволяет не только повысить эффективность репигментации, но и минимизировать риски системных побочных эффектов. Важно продолжать исследование путей снижения риска развития метаболических нарушений и обеспечить раннюю диагностику и коррекцию эндогенных нарушений, ассоциированных с витилиго.

При стабильных, резистентных к консервативной терапии формах заболевания, важным этапом могут стать методы хирургической трансплантации меланоцитов. Согласно данным российских экспертов, к ним относятся как тканевые (трансплантация эпидермальных пузырей, мини-трансплантаты), так и более перспективные клеточные методы: трансплантация некультивированной или культивированной аутологичной суспензии меланоцитов [39][40]. Эффективность последних в российской практике подтверждена: трансплантация культивированных меланоцитов позволила добиться полной репигментации у 81% пациентов [40]. Перспективным направлением является применение мезенхимальных стромальных клеток, обладающих иммуномодулирующим потенциалом. Ключевым условием для любого хирургического вмешательства является стабильное течение витилиго (не менее 12 месяцев) и компенсация сопутствующих эндокринных нарушений, что подчеркивает необходимость междисциплинарного подхода [39][40].

Важно продолжать исследование путей снижения риска развития метаболических нарушений и обеспечить раннюю диагностику и коррекцию эндогенных нарушений, ассоциированных с витилиго.

Настоящая работа служит ориентиром для последующего обсуждения и анализа текущих достижений в изучении ассоциации витилиго с эндокринными заболеваниями, способствует привлечению внимания профессионалов к комплексной оценке состояния пациентов с витилиго и стимулирует продолжение научно-исследовательской деятельности в данном направлении

## ДОПОЛНИТЕЛЬНАЯ ИНФОРМАЦИЯ

Источники финансирования. Работа выполнена по инициативе авторов без привлечения финансирования.

Конфликт интересов. Авторы декларируют отсутствие явных и потенциальных конфликтов интересов, связанных с содержанием настоящей статьи.

Участие авторов. Все авторы одобрили финальную версию статьи перед публикацией, выразили согласие нести ответственность за все аспекты работы, подразумевающую надлежащее изучение и решение вопросов, связанных с точностью или добросовестностью любой части работы.
